# Ecoinformatic Analysis of the Gut Ecological Diversity of Wild and Captive Long-Tailed Gorals Using Improved ITS2 Region Primers to Support Their Conservation

**DOI:** 10.3390/microorganisms11061368

**Published:** 2023-05-23

**Authors:** Chang-Eon Park, Bum-Joon Cho, Min-Ji Kim, Min-Chul Kim, Min-Kyu Park, Jang-Ick Son, Hee-Cheon Park, Jae-Ho Shin

**Affiliations:** 1 Department of Applied Biosciences, Kyungpook National University, Daegu 41566, Republic of Korea; aeonrapt@knu.ac.kr (C.-E.P.); tbd01188@knu.ac.kr (M.-J.K.);; 2 Institute of Ornithology, Ex Situ Conservation Institution Designated by the Ministry of Environment, Gumi 39105, Republic of Korea; 3 Wildlife Union, Donghae 25802, Republic of Korea; hl2xsg@hanmail.net; 4 NGS Core Facility, Kyungpook National University, Daegu 41566, Republic of Korea; 5 Department of Integrative Biotechnology, Kyungpook National University, Daegu 41566, Republic of Korea; 6 Northern Conservation Center, National Park Institute for Wildlife Conservaation, Korea National Park Service, Inje 24607, Republic of Korea

**Keywords:** conservation biology, fungal ITS2 region primers, in silico primer improvement

## Abstract

Ex situ conservation is used to protect endangered wildlife. As captive and wild long-tailed gorals are known to be similar, individuals under ex situ conservation can be reintroduced into nature. However, there is no appropriate indicator to evaluate them. Here, we amplified the internal transcribed spacer 2 (ITS2) region and compared the gut ecological information (eco-information) of captive and wild long-tailed gorals. We validated the existing ITS86F and ITS4 universal primers using reference sequences of the National Center for Biotechnology Information (NCBI) and improved their matching rates. We compared the gut eco-information of captive and wild long-tailed gorals obtained through experiments using the improved primer pair and found that the gut ecological diversity of captive gorals was low. Based on this, we suggested that the gut eco-information can be used as an evaluation index before reintroducing captive long-tailed gorals. Furthermore, we identified four plant types from the gut eco-information of wild long-tailed gorals, which can be the additional food sources to enhance the reduced intestinal ecological diversity of the captive animals.

## 1. Introduction

Currently, endangered wildlife species (EWS) can be protected using methods including in situ and ex situ conservation [1,2], habitat restoration [3], and reintroduction [4]. In situ conservation includes protecting the ranges of habitat of EWS and artificially supplying them with food when natural sources are scarce. Habitat restoration refers to artificially creating potential habitats for EWS that do not currently live there. Ex situ conservation involves maintaining the minimum population of EWS to prevent extinction in places other than their habitats. Reintroduction involves returning the conserved individuals to a stable habitat.

Previously, camera traps have been installed to analyze the survival of EWS within the habitat [5]. The health of ex situ conserved EWS can be evaluated via blood collection, weight measurement, and external morphological factors [6].

However, advances in next-generation sequencing (NGS) have revealed that the gut microbiome is closely related to health and can be used to evaluate the health of EWS [7]. As it is challenging to directly observe endangered wildlife in their natural state, the gut microbiome can be analyzed using their feces to evaluate their conditions scientifically.

In addition, as science and technology continues to advance and scientists’ understanding of information broadens, ecoinformatics is emerging as a field that comprehensively covers all ecological information that can be obtained [8,9,10]. Therefore, it is necessary to apply ecoinformatics to convergence studies of wildlife ecology and microbial ecology. The long-tailed goral (*Naemorhedus caudatus*), a representative EWS, was selected as a research subject because it has undergone both in situ and ex situ conservation. This enables the analysis and comparison of the gut microbiomes of captive and wild animals.

Although long-tailed gorals are mainly herbivores [11], they are often observed licking rocks [12] or soil to consume minerals. Therefore, it is likely that they consume several moss and soil microorganisms. Additionally, specific plants such as mulberry leaves are mainly supplied when breeding for ex situ conservation. Based on this, besides the bacterial community, an efficient method is required to evaluate the fungal communities, ingested plants, and mosses in the gut microbiome of these animals.

Globally, bacterial communities have been the focus of research; however, the importance of studying fungal communities, particularly in wild animals, is progressively garnering attention. Although wild animals play a significant ecological role in consuming and transporting fungi in large quantities, studies have focused on the bacterial community in the gut biome while neglecting other areas [13]. Therefore, our aim is to provide information on the ecological diversity of the mycobiome (living) and ingested plants and mosses (dead communities) that have been poorly studied thus far.

Further, amplification of the internal transcribed spacer 2 (ITS2) region, commonly present in plants [14], mosses [15], and fungi [16], provides a cost-effective solution to obtain more information in a single experiment. However, despite the progress in the research on the amplification of the 16s rRNA region for analyzing existing bacterial communities [17], there is insufficient information regarding the amplification of the ITS2 region, which is mainly used to analyze the fungal community. There is also insufficient information on the matching rate of the primers used to amplify the ITS2 region with the highly variable ITS2 region in the real environment. Therefore, research on these areas is urgently required.

In this study, our first hypothesis was that NGS using the ITS2 region primer pair would yield maximum information on the intestinal ecology of long-tailed gorals, which are herbivores, in a single experiment. Our second hypothesis was that the universal ITS2 region primer pair would not match the actual environment owing to insufficient research, and if improvement was required, we tried to improve the universal ITS2 region primer pair and use it. Additionally, given the recognition of the importance of ecoinformatics research for conservation biology [18], we aimed to provide more ecological information by combining ecoinformatics with our two hypotheses and conservation biology studies of EWS. From this, we revealed that studying the gut ecological information of long-tailed gorals is helpful for their conservation.

## 2. Materials and Methods

### 2.1. Fecal Sample Collection

We collected the fecal samples of wild long-tailed gorals from several locations in Korea, including Seoraksan, Odaesan, Taebaeksan, Woraksan, and Juwangsan national parks, Wangpicheon Conservation Area, and an unnamed mountain in Gagok-myeon, Samcheok. The fecal samples of captive long-tailed gorals were collected from the Northern Conservation Center of the National Park Service and Association of Korean Goral Conservation. Fecal samples from wild long-tailed gorals were collected from locations where they defecate within their natural habitat. Fecal samples from captive long-tailed gorals were obtained from ex situ conservation sites (Table 1). Only fresh fecal samples not contaminated with soil were selected, and sterile nitrile gloves were used for collection. New sterile nitrile gloves were exchanged for each collection, and the collected samples were placed in sterilized zipper bags. Samples in sterile zipper bags were immediately placed in an insulated bag and safely transported to the laboratory without damage where they were stored in a −80 degree of Celsius deep freezer refrigerator until DNA extraction experiments began.

### 2.2. Evaluating and Improving the Performance of the In Silico ITS2 Region Primers

To evaluate the performance of the existing universal primers, ITS86F and ITS4 [19], all available sequences (11,892 sequences at the time of download) were downloaded from the ITS RefSeq Nucleotide sequences [20] of the National Center for Biotechnology Information (NCBI) [21]. The downloaded sequences were aligned with a Python script [22] and then analyzed. Based on the results, we obtained improved CEP-ITS86F and CEP-ITS4 primer pairs and primers for amplifying trace fungal DNA.

### 2.3. Gut Eco-Information Analysis: Fungi and Undigested DNA of Viridiplantae

Fecal DNA was extracted from 250 mg of homogenized fecal solution using the QIAamp PowerFecal Pro DNA Kit (Qiagen, Hilden, Germany) [23,24,25] following the manufacturer’s protocol.

The extracted total DNA was used as a template for amplifying the ITS2 region of the nuclear ribosomal DNA (nrDNA). The extracted total DNA was amplified with the improved ITS2 primer pair (CEP-ITS86F, CEP-ITS4). Deionized–distilled water was used as a negative control, and DNA of the fungal strain *Botrytis cinerea* KACC 40573 was used as a positive control. Then, the barcoded sequences were added to construct a sequencing library.

Sequencing library PCR reaction was performed in a 25 μL reaction: 12.5 μL of 2X EmeraldAmp PCR master Mix (Takara Biotechnology Co., Ltd., Shiga, Japan) [26], 0.1 μM (final conc.) of each primer, and 1 ng of template. The PCR reactions were performed with the following temperature program: 5 min of pre-denaturation at 95 °C, followed by seven cycles of denaturation (95 °C for 30 s), annealing (55 °C for 30 s), extension (72 °C for 30 s), 25 cycles of denaturation (95 °C for 30 s), annealing and extension (72 °C for 1 min), and a final extension at 72 °C for 5 min. The prepared libraries were diluted to the same molecular weight, transferred to an Eppendorf tube, and purified using AMPure XP beads (Beckman Coulter, Brea, CA, USA) [27] to complete the final library.

The final library was diluted to 8 pM, and emulsion PCR was performed using Ion Sphere Particles (ISPs) with the Ion OneTouch System II (Thermo Fisher Scientific, Waltham, MA, USA). This was followed by enrichment for template-positive ISPs using Dynabeads MyOne Streptavidin C1 beads (Thermo Fisher Scientific, Waltham, MA, USA).

The final library after emulsion PCR and the purification process was loaded onto an Ion 318 Chip Kit v2 BC (Thermo Fisher Scientific, Waltham, MA, USA) and sequenced using the Hi-Q View Sequencing Kit and the Ion Torrent Personal Genome Machine (Thermo Fisher Scientific, Waltham, MA, USA) [28] at the Next-Generation Sequencing Center of Kyungpook National University (KNU NGS Center).

Torrent Suite v 5.0 (Thermo Fisher Scientific, Waltham, MA, USA) software was used to trim the adapter sequences. Low-quality reads were removed so that the Phred quality score was greater than 30 from the sequenced data. The primer-trimmed files were then imported into Quantitative Insights Into Microbial Ecology 2 (QIIME2) (version 2023.2) software [29] in Casava 1.8 single-end demultiplexed format for further processing using different algorithms (implemented in QIIME2), which was also run using the 1% distance (99%) reference sequence from the UNITE QIIME release for Eukaryote 2 (version 2022.11.29) [30] to analyze gut eco-information.

The sequencing reads were analyzed using the dada2 plugin in QIIME2 to generate amplicon sequence variants (ASVs). A trim length of 350 base pairs was used, and at least 1 read was required to pass filtering. ASVs with an abundance of <0.1% of the mean sample depth were then removed from analysis.

The output files from QIIME2 were converted to phyloseq objects using the phyloseq and qiime2r packages in R, which were then used for subsequent analysis with ggplot2. The bar chart was generated using the microbiome package’s plot_composition function with options for 1/100 detection and 10–12/100 prevalence. Alpha diversity was visualized using the plot_richness function of the phyloseq package, and statistical significance was calculated using the ggsignif package. For beta diversity, the Aitchison and Bray–Curtis distances were analyzed with the microViz package, and side box plots were created with the ggside package. Heatmap analysis was performed at the log10 level using the microbiomeutilities package. We also recorded the organisms presumed to be food sources for long-tailed gorals [31,32,33,34,35,36,37,38,39,40,41].

## 3. Results

### 3.1. In Silico Improving the Fungal ITS2 Region Primers

By aligning the ITS region sequences downloaded from NCBI’s ITS RefSeq Nucleotide sequence records, we obtained 9695 and 2197 sequences matching the ITS86F and ITS4 regions, respectively. Of these, we could amplify 7700 (approximately 79.42%) and 2058 (approximately 93.67%) of the existing universal ITS86F and ITS4 primers, respectively (Table 2).

These two universal primer pairs were improved to amplify over 92% of the reference sequences. We also created two forward and two reverse primers to amplify the fungal sequences that are difficult to obtain with the existing universal primers.

The range of melting temperatures (Tm) for primer pairs with degenerated codes is shown in Table 3.

### 3.2. Gut Eco-Information: Fungi and Undigested DNA of Viridiplantae

At the phylum level, two phyla predominated in the gut of captive long-tailed gorals, with Ascomycota and Anthophyta accounting for approximately 79.6% and 17.48%, respectively. The two phyla that dominated the gut of wild long-tailed gorals were Ascomycota and Basidiomycota, accounting for approximately 90.6% and 7.75%, respectively.

**Figure 1:**
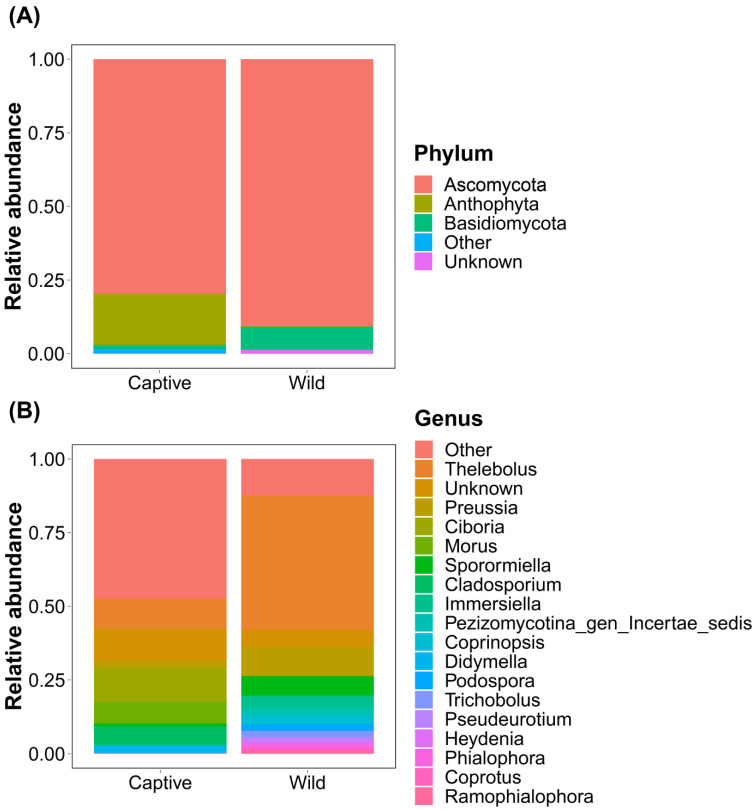
Bar charts showing the relative abundance of total gut ITS2 sequences classified at the (**A**) phylum and (**B**) genus levels.

At the genus level, the gut of captive long-tailed gorals predominantly comprised five genera, including *Ciboria* (approximately 11.56%), *Thelebolus* (approximately 10.25%), *Morus* (approximately 7.4%), *Cladosporium* (approximately 6.42%), and *Didymella* (approximately 2.59%). The five genera that dominated the gut of wild long-tailed gorals were *Thelebolus* (approximately 45.61%), *Preussia* (approximately 9.77%), *Sporormilla* (approximately 6.48%), *Immersilla* (approximately 3.98%), and *Pezizomycotina_gen_Incertae_sedis* (approximately 2.82%) (Figure 1).

We classified 154 ASVs belonging to the kingdoms Fungi and Viridiplantae from captive long-tailed gorals, 11 of which were classified as phylum Anthophyta from Viridiplantae. The nine ASVs classified from wild long-tailed gorals belonged to the phylums Anthophyta (6 ASVs) and Chlophyta (3 ASVs) from Viridiplantae. In the wild types, 23 ASVs were obtained by combining fungi and Viridiplatae, and the ASVs from wild long-tailed gorals were significantly higher than those from captive long-tailed gorals.

### 3.3. Alpha Diversity

**Figure 2:**
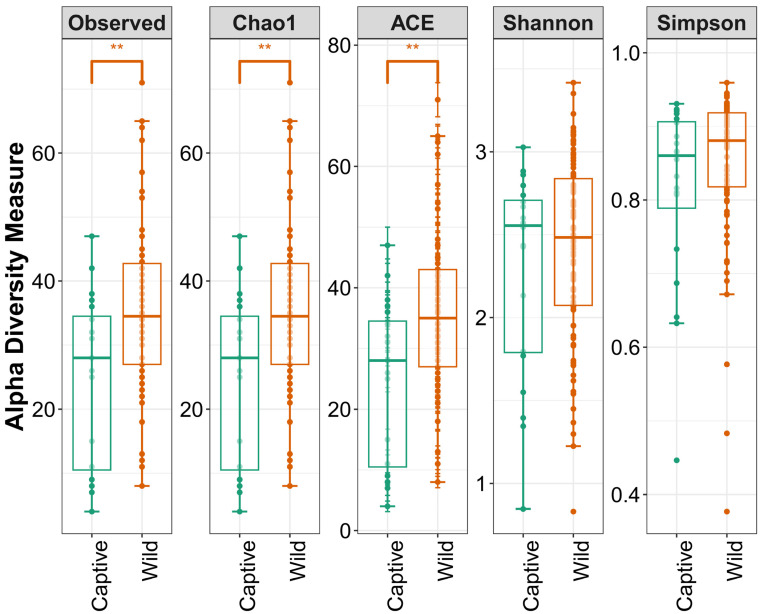
Gut alpha diversity of captive and wild long-tailed gorals. ** *p* < 0.01.

The gut alpha diversity between captive and wild long-tailed gorals was compared using five indices (observed, Chao1, ACE, Shannon, Simpson). Except for the Shannon index, all four indices were higher in the wild long-tailed gorals than in the captive long-tailed gorals. At the *p* < 0.01 level, there was a statistically significant difference in the alpha diversity between captive and wild long-tailed gorals in the three indices (observed, Chao1, ACE) (Figure 2).

### 3.4. Beta Diversity

**Figure 3:**
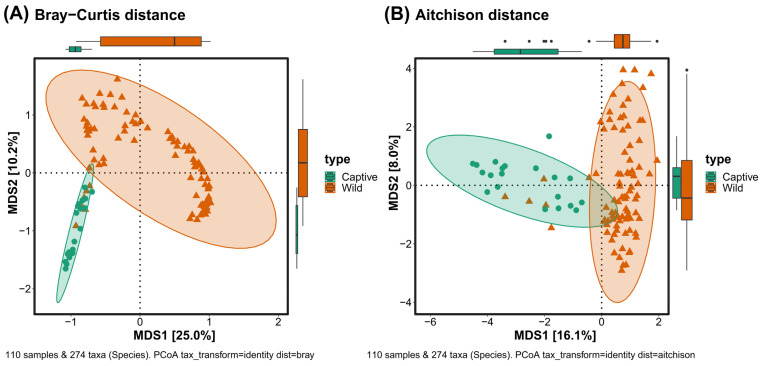
Beta-diversity plots calculated by (**A**) Bray–Curtis and (**B**) Aitchison distance to show the difference in eco-information between captive and wild long-tailed gorals.

Beta diversity was measured using a multidimensional scaling method using Bray–Curtis and Aitchison distances. In the plot calculated with the Bray–Curtis distance, there was a difference between captive and wild long-tailed gorals in both MDS1 and MDS2, while in the plot with the Aitchison distance, there was a difference only in MDS1. In both plots, some of the wild long-tailed gorals were close to their captive counterparts (Figure 3).

### 3.5. Heatmap Analysis

**Figure 4:**
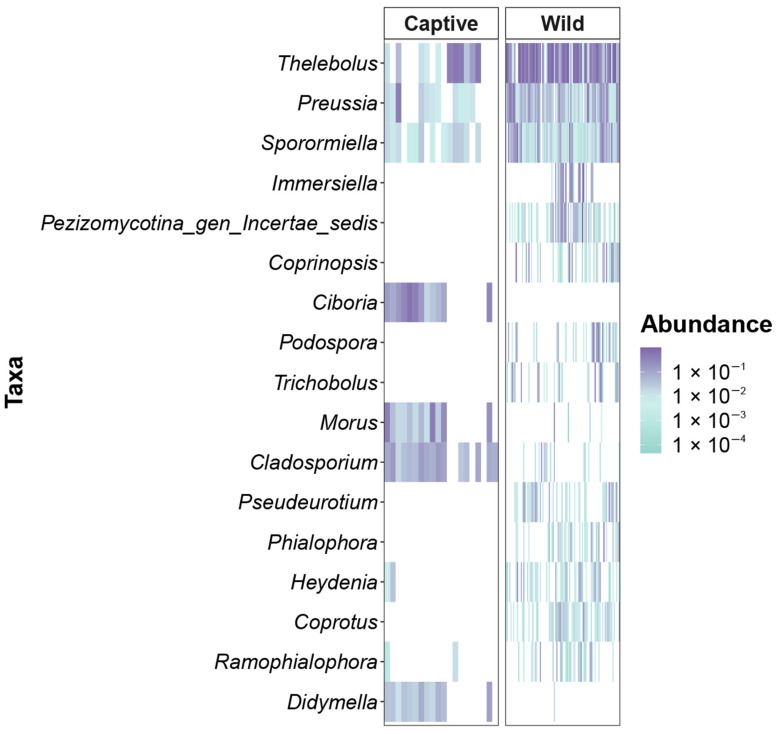
Heatmaps, scaled to the log10 level, illustrating differences in abundance between captive and wild long-tailed gorals.

The heat map analysis revealed 17 genera that were representatively different in captive and wild long-tailed gorals. While the captive long-tailed gorals were represented by the genera *Ciboria*, *Morus*, *Cladosporium*, and *Didymella*, the wild long-tailed gorals were represented by *Thelebolus*, *Preussia*, *Sporormiella*, *Immersiella*, *Pezizomycotina*_gen_Incertae_sedis, *Coprinopsis*, *Podospora*, *Trichobolus*, *Pseudeurotium*, *Phialophora*, *Heydenia*, *Coprotus*, and *Ramophialophora*. Although *Thelebolus*, *Preussia*, and *Sporormiella* appeared frequently in both captive and wild long-tailed gorals, there were quantitative differences. Furthermore, the genera *Immersiella*, *Pezizomycotina*_gen_Incertae_sedis, *Coprinopsis*, *Ciboria*, *Podospora*, *Trichobolus*, *Pseudeurotium*, *Phialophora*, *Heydenia*, and *Coprotus* appeared only in one of the two phenotypes, showing a stark difference (Figure 4).

### 3.6. Undigested DNA of Viridiplantae

The gut eco-information revealed the presence of Viridiplantae and the DNA from plants and microalgae. At the phylum level, two phyla, Anthophyta and Chlorophyta, were observed. At the class level, three classes of Eudicotyledonae, Chlorophyceae, and Trebouxiophyceae appeared. At the order level, seven orders of Fabales, Fagales, Malphighiales, Malvales, Rosales, Sphaeropleales, and Prasiolales were seen, whereas seven families were found: Fabaceae, Juglandaceae, Salicaceae, Malvaceae, Rosaceae, Radiococcaceae, and Stichococcaceae at the family level. At the genus level, nine genera were represented: *Maackia*, *Juglans*, *Salix*, *Tilia*, *Morus*, *Prunus*, *Rubus*, *Coenochloris*, and *Pseudostichococcus*. At the species level, there was *Maackia amurensis*, *Juglans hopeiensis*, *Salix heteromera*, and *Tilia* sp., *Tilia paucicostata* 1, *Tilia paucicostata* 2, *Morus alba* 1, *Morus alba* 2, *Prunus* sp. 1, *Prunus* sp. 2, *Prunus serrulata*, *Rubus microphyllus*, *Coenochloris* sp., *Pseudostichococcus monallantoides*, and *Pseudostichococcus* sp. Overall, we identified 13 plant and three microalgae types based on their ASV. Therefore, the nucleotide sequences for these fifteen ASVs were revealed (Table 4).

## 4. Discussion

### 4.1. Validating and Improving the Fungal ITS2 Region Primers

To the best of our knowledge, this is the first study to evaluate the matching rates of fungal ITS2 region primers compared to the reference sequences from a specific database. While our research was based on the fungal database, other databases can also be used similarly for studying other organisms.

Based on these universal ITS2 region primer sequences, recent studies have used primers with improved 2-mers, such as 5′-GTGARTCATCGARTCTTTGAA-3′ (modified with degenerate code R) under the name of gITS86F. However, the details about how this primer was improved are unclear. Although the modified primers were mentioned, they were cited to the first universal primer paper before modification [42,43,44].

As database research is continuously being conducted worldwide [45], future research needs to be conducted to verify what percentage of the database sequences can be amplified by universal primers and further improve these primers. As scientific technology continues to advance, the accumulation of data will increase, making it necessary to regularly analyze databases, validate universal primers, and improve them. Researchers studying genetic regions for which a database already exists are advised to use previously identified universal primers only after directly verifying their performance rather than relying solely on the contents of previous studies.

Our experiments focus on improving the matching rate of universal ITS2 primer pairs on the reference database. Further research is needed to see how much the matching rate for the real environment improves when an improved new primer pair and an improved existing primer pair are compared experimentally.

Our research results demonstrate that misunderstandings about the actual environment can occur if researchers blindly follow previous studies without conducting in-depth methodological confirmation. This highlights the need to break the stereotype that previous research is always perfect.

### 4.2. Gut Eco-Information of Long-Tailed GORALS

#### 4.2.1. Gut Ecological Diversity

Thus far, only two previous research papers have reported the gut microorganisms of long-tailed gorals in Korea. Regarding gut microbiome research, the only biogeographic study on wild long-tailed gorals was completed by our team [46]. To facilitate in situ and ex situ conservation of long-tailed gorals, it is essential to understand their rich gut eco-information, which has not been shown yet. To our knowledge, this study is the first to compare the gut eco-information, including the microbiome of the two types of long-tailed gorals.

We found that the genus *Thelebolus* was the most dominant in Korean wild long-tailed gorals. *Thelebolus* is known to be dispersed by animals [47], and it is closely related to wild animals and dominant in some ruminants [48]. Considering that it makes up nearly half of the sequences obtained from the gut eco-information of wild long-tailed gorals, it can be seen as evidence that wild long-tailed gorals are excellent *Thelebolus* dispersers that have a significant impact on the ecology and evolution of this genus of soil fungus. We also found an approximate 35% reduction in the abundance of *Thelebolus* in captive long-tailed gorals, which suggests that long-tailed gorals have lost their role as *Thelebolus* dispersers in the wild due to being bred under human interference. Our findings highlight the importance of understanding the ecological information to conserve the role of long-tailed gorals in ecosystem cycles.

We analyzed the gut ecological diversity, including the gut biodiversity and environmental DNA (eDNA) [49,50,51] diversity from plants ingested by the long-tailed gorals. From these results, we found that the gut ecological diversity of long-tailed gorals under ex situ conservation was statistically significantly lower than those living in their native habitat. The low diversity of intestinal microbes in captive long-tailed gorals can be attributed to their limited food source. The captive long-tailed gorals were mainly fed with mulberry leaves, reducing gut ecological diversity. However, the higher gut eco-biodiversity of wild long-tailed gorals is due to various activities such as constantly avoiding predators [52] and consuming several food sources from different regions. Although high gut microbiome diversity is associated with human health [53], it has not yet been elucidated in long-tailed gorals. In general, captive animals in zoos or conservation institutions live longer than wild animals in nature [54]. While long-tailed gorals must look physically healthy and live long, part of the goal of ex situ conservation is to reintroduce them into the wild. If the reintroduction is conducted when the gut conditions of captive long-tailed gorals are significantly different from their wild counterparts, they will need more time to adapt [55].

Therefore, to enable the quick adaptation of long-tailed gorals reintroduced into the wild after ex situ conservation, it is necessary to analyze their gut ecodiversity and implement the reintroduction after their gut condition is as close as possible to the wild type. As the body shape, weight, and blood parameters are indistinguishable in captive and wild long-tailed gorals, their gut eco-information, which is significantly different, should be used as an indicator for reintroduction.

#### 4.2.2. Food Sources of Long-Tailed Gorals from Gut Eco-Information

Based on the gut eco-information, plants such as *Maackia amurensis* (Amur *maackia*), *Morus alba* (white mulberry), *Prunus* sp., and *Rubus microphyllus* were found in the guts of wild long-tailed gorals, which might enhance the gut ecological diversity of captive long-tailed gorals. Although these four plant types can be initially used to keep the gut ecological diversity of captive long-tailed gorals similar to the wild type, we need to continuously collect the gut eco-information of wild long-tailed gorals and accumulate eco-information on the plants they consume.

Our study is the first to compare wild and captive Korean long-tailed gorals and provide information about the plants they consume. From this information, the food sources of captive long-tailed gorals selected for reintroduction projects can be diversified.

#### 4.2.3. Efforts to Reintroduce Gorals in the Future

To use gut eco-information as an indicator for the reintroduction of captive animals into the wild, a three-stage research approach is needed. First, it is necessary to continuously accumulate gut ecology information from both captive and wild endangered species to better understand their ecology, and gut eco-information associated with natural settings and those involving human intervention must be distinguished. Second, a study of the composition of DNA from species living in the gut and environmental DNA corresponding to the plants consumed as food sources is needed. The ITS2 region could be used to study two distinct DNAs simultaneously. Third, captive animals should be provided with the plants consumed by wild animals, accumulated in the second step, to create a more natural gut ecology.

According to the Korean Ministry of Environment, there are over 2000 wild long-tailed gorals living in Korea. As the long-tailed gorals population had drastically decreased to 700 due to natural disasters in the past, more conservation effort is required. When reintroducing captive animals into the wild, we should minimize the process of adaptation for their convenience by providing a variety of food rather than continuously supplying only a few food types.

## 5. Conclusions

As a comparative analysis of the gut eco-information of captive and wild long-tailed gorals in Korea has not been reported yet, our study is the first to provide this information. We validated and in silico improved the matching rates of universal ITS2 primers, and using this improved primer pair, we compared the differences in the gut eco-information of captive and wild long-tailed gorals.

Finally, we suggest that the eco-information should be evaluated before reintroducing captive long-tailed gorals into nature to overcome their low gut ecological diversity. Moreover, we also identified diet plants from the gut eco-information of wild long-tailed gorals, which can be fed to captive long-tailed gorals to enhance their gut ecological diversity.

## Figures and Tables

**Table 1:** Information on fecal samples collected by region.

Regions	No. of Samples	Type of Samples
Seoraksan National Park	26	Wild
Odaesan National Park	8	Wild
Taebaeksan National Park	3	Wild
Woraksan National Park	9	Wild
Juwangsan National Park	2	Wild
Wangpicheon Conservation Area	14	Wild
Unnamed mountain in Samcheok	28	Wild
Northern Conservation Center	11	Captive
Association of Korean Goral Conservation	9	Captive
Total	110	

**Table 2:** Top five ITS86F and ITS4 primer region sequences within the NCBI reference sequences.

Regions	Sequences	Counts	Proportions (%)
ITS86F	GTGAATCATCGAATCTTTGAA	7700	79.4224
	GTGAATCATCGAGTCTTTGAA	888	9.1594
	GTGAGTCATCGAATCTTTGAA	253	2.6096
	GTGAATCATTGAATCTTTGAA	108	1.1140
	GTGAACCATCGAATCTTTGAA	96	0.9902
ITS4	TCCTCCGCTTATTGATATGC	2058	93.6732
	CCTCCGGCTTATTGATATGC	16	0.7283
	CCTCCCGCTTATTGATATGC	12	0.5462
	TCCTCTGCTTATTGATATGC	10	0.4552
	TCCTCCGCTGACTGATATGC	8	0.3641

**Table 3:** Sequences of developed primers and matching rates with NCBI’s reference sequences.

Primers	Sequences	Melting Temperature (°C)	Matching Rate (%)
CEP-ITS86F	GTGARTCATYGARTCTTTGAA	53–59	92.3053
CEP-ITS86F-GCG	GCGARTCATCGARTCTTTGAA	58–62	0.9593
CEP-ITS86F-CTG	CTGAATCATCRAATYTTTGAA	51–55	0.0619
CEP-ITS4-	TCCTCYGCTKAYTGATATGC	56–64	94.8111
CEP-ITS4-CCT	CCTYCSGCTTATTGATATGC	58–61	1.3655
CEP-ITS4-TCT	TCTTCYGCTTATTGATATGY	52–57	0.3186

**Table 4:** Fifteen Viridiplantae ASVs in the guts of captive and wild long-tailed gorals.

Kingdom	Phylum	Class	Order	Family	Genus	Species
Viridiplantae	Anthophyta	Eudicotyledonae	Fabales	Fabaceae	*Maackia*	*amurensis ^c, w^*
		Eudicotyledonae	Fagales	Juglandacea	*Juglans*	*hopeiensis ^c^*
		Eudicotyledonae	Malpighiale	Salicaceae	*Salix*	*heteromera ^c^*
		Eudicotyledonae	Malvales	Malvaceae	*Tilia*	sp. *^c^*
		Eudicotyledonae	Malvales	Malvaceae	*Tilia*	*paucicostata* 1 *^c^*
		Eudicotyledonae	Malvales	Malvaceae	*Tilia*	*paucicostata* 2 *^c^*
		Eudicotyledonae	Rosales	Moraceae	*Morus*	*Morus alba* 1 *^c, w^*
		Eudicotyledonae	Rosales	Moraceae	*Morus*	*Morus alba* 2 *^c, w^*
		Eudicotyledonae	Rosales	Rosaceae	*Prunus*	sp. 1 *^c, w^*
		Eudicotyledonae	Rosales	Rosaceae	*Prunus*	sp. 2 *^c, w^*
		Eudicotyledonae	Rosales	Rosaceae	*Prunus*	*serrulata ^c^*
		Eudicotyledonae	Rosales	Rosaceae	*Rubus*	*microphyllus ^w^*
	Chlorophyta	Chlorophycea	Sphaeropleales	Radiococcaceae	*Coenochloris*	sp. *^w^*
		Trebouxiophyceae	Prasiolales	Stichococcaceae	*Pseudostichococcus*	*monallantoides ^w^*
		Trebouxiophyceae	Prasiolales	Stichococcaceae	*Pseudostichococcus*	sp. *^w^*

*^c^* captive; *^w^* wild.

## Data Availability

The microbiota data used in this study are available in Bioproject PRJNA739827.
